# Predictors and Changes in Paternal Perinatal Depression Profiles—Insights From the DREAM Study

**DOI:** 10.3389/fpsyt.2020.563761

**Published:** 2020-10-29

**Authors:** Susan Garthus-Niegel, Andreas Staudt, Patricia Kinser, Silje Marie Haga, Filip Drozd, Sophie Baumann

**Affiliations:** ^1^Department of Medicine, Faculty of Human Sciences, Medical School Hamburg, Hamburg, Germany; ^2^Department of Child Health and Development, Norwegian Institute of Public Health, Oslo, Norway; ^3^Faculty of Medicine, Institute and Policlinic of Occupational and Social Medicine, Technische Universität Dresden, Dresden, Germany; ^4^School of Nursing, Virginia Commonwealth University, Richmond, VA, United States; ^5^Department for Infant Mental Health, Regional Centre for Child and Adolescent Mental Health, Eastern and Southern Norway, Oslo, Norway

**Keywords:** paternal perinatal depression, depression profiles, latent class analysis, latent transition analysis, DREAM study

## Abstract

In contrast to the large body of research on maternal perinatal depression, *paternal* perinatal mental health has received little attention; and longitudinal studies on paternal perinatal depression, following (expectant) fathers over time, are exceedingly rare. This population-based study aimed to (1) estimate prevalence rates of perinatal depression symptoms among German (expectant) fathers, (2) identify differential profiles of perinatal depression in (expectant) fathers, (3) determine modifiable predictors of latent depression profiles, and (4) estimate how membership in subgroups changes during the perinatal period. Data were derived from the longitudinal cohort study DREAM (Dresden Study on Parenting, Work, and Mental Health), including 1,027 (expectant) fathers responding to the Edinburgh Postnatal Depression Scale (EPDS) during pregnancy and 8 weeks postpartum. Unobserved profiles of paternal perinatal depression and movement between profiles were investigated using latent transition analysis. A number of potential predictors with regard to lifestyle and current life situation were included as covariates. We found that rates of paternal depression symptoms decreased with 9% during pregnancy to 5% at 8 weeks postpartum. Further, four latent depression profiles emerged: most (expectant) fathers did not exhibit any depression symptoms (*not depressed*), whereas some reported mainly the absence of joy (*anhedonic*) and some experienced mainly self-blame and worries (*anxious-worried*). The *depressive* profile was characterized by endorsement to most symptoms of perinatal depression. Perceived social support and relationship satisfaction appeared to be protective against paternal depression symptoms. Differential transitioning or stability patterns in profile membership during the perinatal period were found, whereas the *depressive* profile showed to be the least stable. This prospective population-based cohort study is the first study to identify paternal perinatal depression profiles together with their predictors and changes during the perinatal period. Future research is warranted to examine whether the identified paternal depression profiles have differential outcomes, particularly in the context of person-centered prevention and intervention strategies. Further, longitudinal trajectories of paternal depression ought to be studied, taking into account additional measurement points as well as modifiable risk factors.

## Introduction

The perinatal period is a time involving much emotional turmoil for (expectant) parents. Regarding (expectant) mothers, studies suggest that women are at increased risk for mental health concerns during this life period (1) and report that 10–15% of women experience clinically significant depression symptoms during pregnancy or the postpartum period (2, 3). Recent studies highlight that women are at equal risk of developing depression during pregnancy as after birth (4), and one of the greatest risks factors for the onset of postpartum depression is depression symptoms in pregnancy (5–8). Core symptoms of maternal perinatal depression comprise tearfulness, feelings of hopelessness, inadequacy, guilt, inability to cope with and feel joy over the new baby, agitation and anxiety, loss of appetite, poor concentration and memory, sleep disturbances, fatigue, social isolation, and suicidal ideation (9). Perinatal depression symptoms are a major cause for concern as they directly or indirectly increase maternal morbidity and mortality (10). Indeed, clinically significant depression symptoms are projected to be a leading cause of illness and disability in the world by 2030; further, suicide is currently a major cause of maternal death in developed countries (10, 11). Children of affected women are at increased risk of being born preterm or with low birth weight (12) and are less frequently breastfed (13). Also, women who suffer from perinatal depression are less capable of interacting with their infant in an appropriate and warm manner, such as engaging in important developmental activities with the baby (e.g., playing and talking) which may negatively influence the child's cognitive and socioemotional development (14–16) and the infant's attachment style (17).

In contrast to the large body of research on maternal perinatal depression, *paternal* perinatal mental health has received little attention from researchers and clinicians, and very little is known about paternal perinatal depression (18–21). However, as with their maternal counterparts, fathers appear to be at increased risk of depression in the perinatal period (22–26). While the prevalence rate of depression in men in the general population is ~4.8% (27), a meta-analysis found that the rate of paternal depression between the first trimester and 1 year postpartum was 10.4% (19). This suggests that paternal perinatal depression also represents a significant public health concern (19). As gender roles shift and paternal involvement in childcare is becoming the norm in western societies, fathers' mental health becomes increasingly important for their children (19, 28). Indeed, there is mounting evidence that early paternal depression may have substantial emotional, behavioral, and developmental effects on children (19, 29–31). For instance, longitudinal cohort studies from the United Kingdom have shown that depression symptoms in the father at 2 months postpartum could predict a higher risk of behavioral problems in children at 3.5 years of age (32) and an increased risk of behavioral and conduct disorders, including peer relationship difficulties, by 7 years of age (29, 33). Paternal depression during the perinatal period may resemble maternal perinatal depression (19), although some evidence suggests that depression in men is characterized more often by a high level of general distress (33, 34). Thus far, data on paternal perinatal depression are based on an emerging and inconsistent literature, and longitudinal studies on paternal perinatal depression, following (expectant) fathers over time, are exceedingly rare (19).

Given the clear need for longitudinal studies on paternal perinatal depression, this population-based study aimed to (1) estimate prevalence rates of perinatal depression symptoms among German (expectant) fathers, (2) identify differential profiles of perinatal depression in (expectant) fathers, (3) determine modifiable predictors of latent depression profiles, and (4) estimate how membership in subgroups changes during the perinatal period.

## Materials and Methods

### Study Setting and Design

This investigation is part of the longitudinal cohort Dresden Study on Parenting, Work, and Mental Health (DREAM; DResdner Studie zu Elternschaft, Arbeit, und Mentaler Gesundheit), which prospectively examines the relationship between parental work participation, role distribution, stress factors, and their effects on perinatal outcomes and long-term family mental and somatic health (35). Expectant mothers and their partners were recruited during pregnancy, predominately at information sessions in hospitals and birth preparation courses in and around Dresden, Germany. The DREAM study consists of currently four measurement points, during which questionnaires covering a comprehensive field of physical and mental health outcomes are completed by participants. The measurement points encompass time point one (T1; during pregnancy), and three postpartum assessment waves: time point two (T2) at 8 weeks after the anticipated birth, time point three (T3) at 14 months, and time point four (T4) at 2 years after birth [postpartum follow-up data collection is still ongoing and prolongation into middle childhood planned; for a detailed description of the study see (35)]. The DREAM study has been reviewed and approved by the Ethics Committee of the Faculty of Medicine of the Technische Universität Dresden (No: EK 278062015), and all participants provided their written informed consent to participate in this study. Study data were collected and managed using Research Electronic Data Capture (REDCap), hosted at the “Koordinierungszentrum für Klinische Studien” at the Faculty of Medicine of the Technische Universität Dresden (36, 37). For the purpose of the present study, T1 (during pregnancy) and T2 (8 weeks postpartum) data from 1,027 (expectant) fathers were analyzed.

### Measures

#### Paternal Perinatal Depression

Paternal perinatal depression symptoms were measured by the German version of the Edinburgh Postnatal Depression Scale [EPDS; (38)], which was administered at T1 and T2. The EPDS is the most common scale to screen for symptoms of maternal perinatal depression (39) and has been validated in numerous studies. It has been shown to be a valid instrument for identifying probable major depression in postnatal fathers (21, 34). The EPDS is a 10-item self-report instrument, scored on a four-point scale [0–3; (38, 40)], with scores ranging from 0 to 30 whereby higher scores suggest higher levels of depression symptoms.

The 10 EPDS items were used as manifest indicators of latent paternal depression profiles. The items were dichotomized in order to represent approval or refusal of each symptom. Dichotomized items were chosen over the original scale and a three-categorical solution because very few individuals scored in the two highest categories of the four-point EPDS scale. Moreover, using the original scale would add substantial complexity to the identification and distinction of latent profiles, not least because the response options do not seem to be equidistant. Due to the item-specific labels of the EPDS, the cut-off, above which a symptom was regarded as endorsed, had to be chosen for each item individually. The dichotomization was either carried out between response options 0–1 and 2–3 (EPDS items 3–8) or between 0 and 1–3 (EPDS items 1, 2, 9, and 10).

For the purpose of sample description, the sum score of the EPDS was used. The prevalence of paternal perinatal depression is reported using the most common cut-off scores of ≥10 to indicate minor depression and ≥12 to indicate major depression (34, 38, 40). For all latent variable models, single dichotomized EPDS items were used (see section Statistical Analysis).

#### Covariates

Socio-demographic characteristics, relationship factors, and health behaviors were assessed at T1. Current paternal age was calculated from (expectant) fathers' date of birth and the date they answered the T1 questionnaire. (Expectant) fathers were asked to indicate the number of children below the age of 14 living in the same household and their highest general educational degree, which was condensed into two categories (<12 years/12 or more years of school education).

Perceived social support was measured with the short version of the Social Support Questionnaire [F-SozU-14; (41)]. The short version of the Partnership Questionnaire [PFB-K; (42)] was used to assess relationship satisfaction. For both questionnaires, the sum score was calculated, with higher scores indicating higher levels of perceived social support and relationship satisfaction, respectively. One item was used to assess the distribution of housework between the two partners. The original scale ranged from 0 (“I do everything”) to 10 (“My partner does everything”) and was recoded so that 0 represents an equal distribution of household duties (five on the original scale) and higher values (deviations from 5) indicate an inequitable distribution to either side.

(Expectant) fathers' Body Mass Index (BMI) was calculated from their indicated height and weight. Self-reported alcohol consumption was measured with a quantity-frequency index, resulting in four categories: 0, 1–2, 3–7, or 8 or more alcoholic standard drinks consumed per week. Smoking was assessed with three categories: never smoked, former smokers or current smokers. Expectant fathers were asked to indicate how often they engage in physical activity per week (e.g., brisk walking, going to work by bicycle, sports): less than once per week, 1–2 times per week, or 3 times or more per week.

Three variables from the respective expectant mothers' T1 questionnaire were included as covariates: the expectant mothers' age, her highest educational degree, and her EPDS sum score.

### Statistical Analysis

To identify paternal perinatal depression profiles, and to estimate predictors and changes in subgroup membership, mixture modeling was used. Descriptive statistics and dropout analyses were done in Stata 14 (43). Logistic regression was used to test if T1 variables predicted non-participation at T2. Latent variable modeling was performed using Mplus version 7.4 (44). All latent variable models were analyzed with a full-information maximum likelihood estimator with robust standard errors, which uses all available data under the assumption of missing at random.

Data were analyzed in consecutive steps following the framework proposed by Ryoo et al. (45). At first, latent paternal perinatal depression profiles were identified using Latent Class Analysis (LCA). In LCA, manifest indicators (i.e., the 10 dichotomized EPDS items) are used to extract unobserved subgroups (latent classes) represented by a categorical latent variable. The aim is to find the number of latent classes that adequately represents the heterogeneity in a study population. The identification of the number of latent paternal depression profile classes was based on T1 data without adjusting for any covariates.

Models with different numbers of latent classes were compared using the Bayesian Information Criterion [BIC; (46)], the Akaike Information Criterion [AIC; (47)], the bootstrapped likelihood ratio test [BLRT; (48)], the class sizes as well as the conceptual meaning and distinctiveness of each latent class. BIC and AIC balance fit and parsimony of latent models, whereas models with smaller values are superior. The BLRT compares a model with *k* latent classes to a model with *k-1* latent classes, whereas statistical significance favors the *k*-class-model. To evaluate clarity in classification, entropy (49) was obtained, with values ≥ 0.80 indicating adequate classification (50). Each model was rerun with a higher number of starting values (1,000 instead of 20) to ensure that the solutions are replicated and not caused by local maxima.

**Figure 1:**
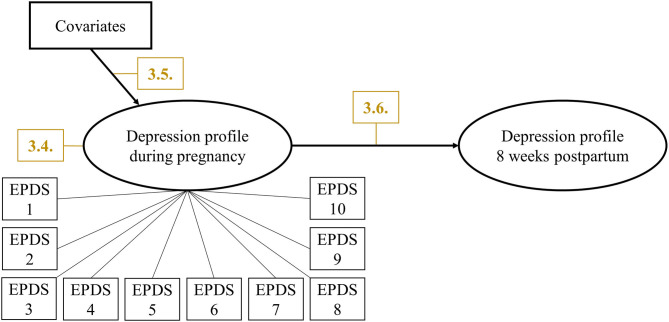
Final LTA modeling predictors and changes in paternal perinatal depression profiles. Manifest indicators for depression profile at 8 weeks postpartum were omitted for clarity. Yellow numbers indicate corresponding sections in Results.

Then, data from T2 were analyzed in a similar manner to determine if the same number of latent classes were found for T1. Changes in latent class membership during the perinatal period from T1 to T2 were estimated using Latent Transition Analysis (LTA). In LTA, latent transition probabilities are estimated that represent the probabilities of changing the latent class from T1 to T2. The prerequisite for LTA is measurement invariance over time which allows to interpret changes in latent class membership as actual changes in paternal depression profiles because the latent classes have the same meaning over time. Measurement invariance was tested by comparing a measurement invariant LTA model in which the thresholds of class indicators were held equal over time with a measurement variant LTA model in which thresholds were allowed to vary over time using BIC values. To evaluate whether the LTA model-implied data fit to the empirical data, bivariate standardized residuals (BSRs) were used, with values below |1.96| indicating good fit. For the final LTA model (Figure 1), covariates were included to predict latent class membership at T1 in a multinomial logistic regression. Results were given as *Odds Ratios* (*OR*) with *95% confidence intervals* (*95%-CI*).

## Results

### Participant Flow

By January 2020, *n* = 1,194 expectant fathers returned the first questionnaire (T1). Those expecting more than one child (*n* = 21), those who returned the questionnaire after birth (*n* = 21), and those who had incomplete data on the covariates (*n* = 125) were excluded. Thus, 1,027 expectant fathers were included into the analysis.

Of the *n* = 1,008 fathers, who had already received the second questionnaire (T2), *n* = 860 (85 %) fathers had returned the completed questionnaire on time. Those who were not reached for T2 (*n* = 145) were less likely to have children below the age of 14 in the household prior to the index baby (OR = 0.52, 95% CI: 0.30–0.90) and were more likely to report former (OR = 1.79, 95% CI: 1.16–2.76) or current smoking (OR = 2.23, 95% CI: 1.36–3.64).

### Sample Characteristics

Baseline sample characteristics are shown in Table 1. The final sample was composed of 1,027 men with a mean age of 32.4 years (*SD* = 4.9). The majority of participants (*n* = 675, 65.7%) had 12 or more years of school education. During pregnancy (T1), the mean EPDS score was 3.9 (*SD* = 3.6). According to the standard EDPS cut-off scores, 50 expectant fathers (5%) screened positive for minor depression (≥10) and 40 (4%) for major depression (≥12). At the postpartum (T2) assessment, the mean EPDS score was 3.5 (*SD* = 3.3). Of those participating in the T1 assessment, 20 (2%) screened positive for minor depression and 22 (3%) for major depression.

**Table 1:** Sample characteristics during pregnancy (T1).

**Variable**	***M*** **(SD)/*****n*** **(%)**	
Age	32.4	(4.9)
**Years of school education**		
<12 years	352	(34.3)
12 or more years	675	(65.7)
**Children below age 14 in household**		
No	839	(81.7)
Yes	188	(18.3)
EPDS score	3.9	(3.6)
**Perinatal depression according to EPDS**		
No depression	937	(91.2)
Minor depression (score ≥ 10)	50	(4.9)
Major depression (score ≥ 12)	40	(3.9)
Body Mass Index	25.6	(4.2)
**Physical activity**		
Less than once a week	290	(28.2)
1–2 times a week	391	(38.1)
3 or more times a week	346	(33.7)
**Smoking status**		
Never smoked	530	(51.6)
Former smoker	310	(30.2)
Current smoker	187	(18.2)
**Alcoholic drinks consumed per week**		
None	208	(20.3)
1–2 drinks	258	(25.1)
3–7 drinks	349	(34.0)
8 drinks or more	212	(20.6)
Perceived social support (F-SozU-14 score)	58.7	(9.08)
Relationship satisfaction (PFB-K score)	20.6	(4.2)
Inequitable distribution of domestic work	1.0	(1.0)
Expectant mother's age	29.9	(3.8)
**Expectant mother's years of school education**		
<12 years	252	(24.5)
12 or more years	775	(75.5)
Expectant mother's EPDS score	5.5	(4.1)

*M, mean; SD, Standard Deviation; EPDS, Edinburgh Postnatal Depression Scale; F-SozU-14, short version of the Social Support Questionnaire; PFB-K, short version of Partnership Questionnaire. N = 1,027*.

### Identification of Latent Depression Profile Classes

Table 2 shows fit indices, entropy values, and class sizes for separate LCA models specifying one to six classes. While AIC decreased with increasing number of classes, BIC and BLRT *p*-values indicated preferred fit for the four-class models. Therefore, we selected the four-class solution for LTA. BIC values indicated preferred fit for the measurement invariant LTA model (BIC = 10,802) over the measurement variant LTA model (BIC = 10,987). Of the 760 BSRs from the measurement invariant LTA, 738 values (97%) were below |1.96|. The largest residual value was 3.86. Entropy for the final LTA model with covariates was 0.84.

**Table 2:** Fit indices for LCA models specifying two to six classes.

		**2-class model**	**3-class model**	**4-class model**	**5-class model**	**6-class model**
**T1: during pregnancy**						
AIC		6,273	6,126	6,042	6,005	6,004
BIC		6,377	6,284	6,254	6,271	6,324
BLRT		*p*-value	<0.001	0.001	0.001	0.104	0.166
Entropy		0.825	0.791	0.835	0.883	0.886
Class size, *n* (%)	class 1	834 (81)	706 (69)	758 (74)	758 (74)	732 (71)
	class 2	193 (19)	281 (27)	143 (14)	151 (14)	157 (16)
	class 3		40 (4)	88 (8)	49 (5)	44 (4)
	class 4			38 (4)	41 (4)	43 (4)
	class 5				28 (3)	39 (4)
	class 6					12 (1)
**T2: 8 weeks postpartum**						
AIC		4,635	4,480	4,410	4,405	4,401
BIC		4,735	4,632	4,615	4,662	4,710
BLRT		*p*-value	<0.001	<0.001	<0.001	0.063	-^*^
Entropy		0.817	0.778	0.807	0.770	0.796
Class size, *n* (%)	class 1	732 (85)	597 (70)	620 (72)	561 (65)	561 (65)
	class 2	128 (15)	244 (28)	123 (12)	167 (20)	178 (21)
	class 3		19 (2)	99 (14)	81 (9)	74 (8)
	class 4			18 (2)	38 (4)	22 (3)
	class 5				13 (2)	13 (2)
	class 6					12 (1)

*AIC, Akaike Information Criterion; BIC, Bayesian Information Criterion; BLRT, Bootstrapped likelihood ratio test*.

* *p-value not trustworthy due to local maxima. T1 LCA model: N = 1,027; T2 LCA model: N = 860*.

### Characteristics of Latent Depression Profile Classes

**Figure 2:**
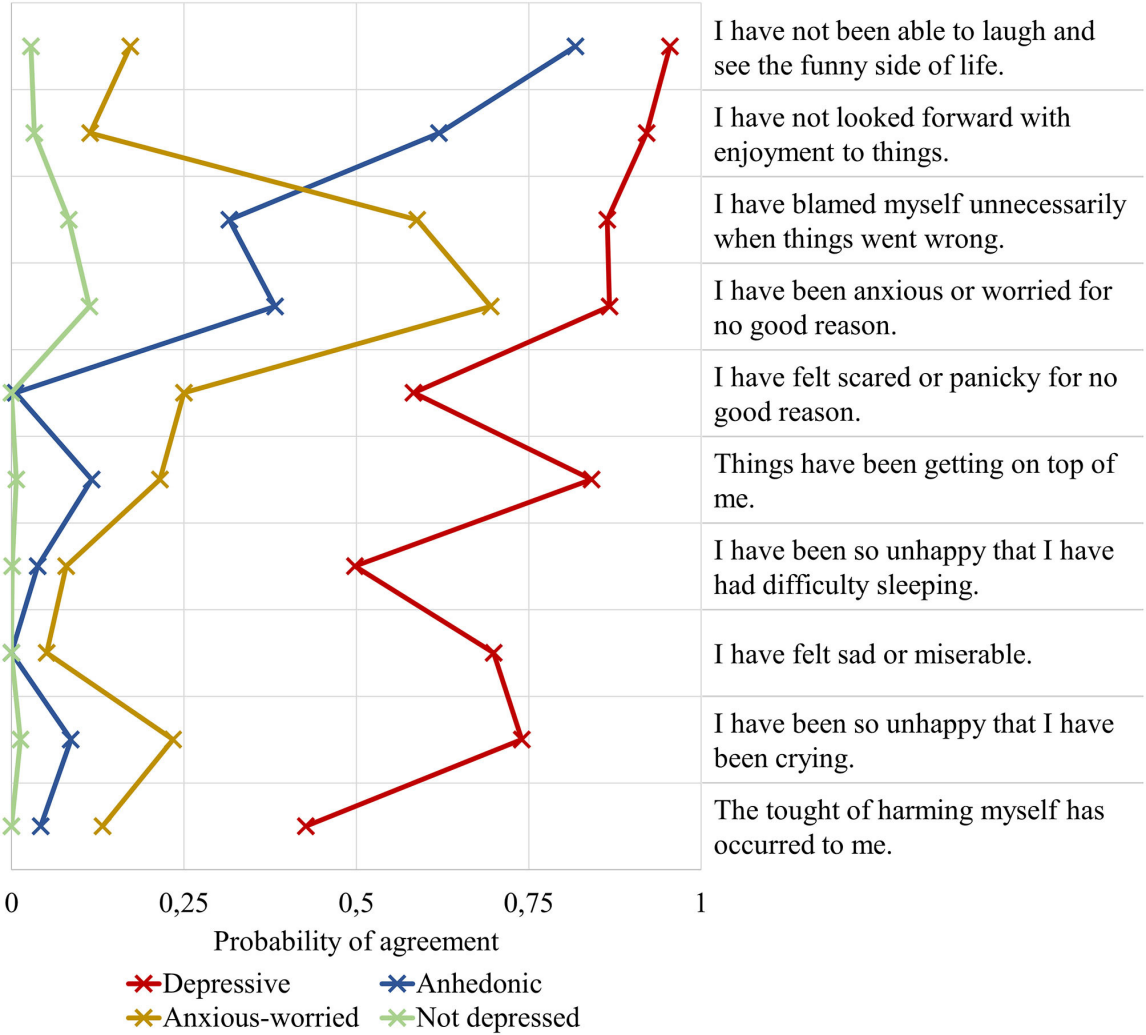
Estimated item-response probabilities by latent depression profile.

The estimated class-specific probabilities of scoring high on the EDPS items for the four-class model are depicted in Figure 2. (Expectant) fathers in the largest class (*n* = 677, 66%) were characterized by near-zero probabilities of scoring high on the EPDS items. This class was labeled *not depressed*. About 17% of the (expectant) fathers (*n* = 178) were allocated to an *anhedonic* class characterized by high probabilities of scoring high on EPDS items 1 (“I have not been able to laugh and see the funny side of things”) and 2 (“I have not looked forward with enjoyment to things”). (Expectant) fathers in the *anxious-worried* class (*n* = 142, 14%) were characterized by high probabilities of scoring high on EPDS items 3 (“I have blamed myself unnecessarily when things went wrong”) and 4 (“I have been anxious or worried for no good reason”). The smallest class (*n* = 30, 3%) consisted of (expectant) fathers with high probabilities of agreeing with all EPDS items. This class was labeled *depressive*.

### Predictors of Latent Depression Profile Class Membership

As shown in Table 3, (expectant) fathers in the *anhedonic* class were less likely to engage in physical activity three or more times vs. once a week (OR = 0.48, 95% CI: 0.27–0.85), reported lower social support (OR = 0.94, 95% CI: 0.92–0.97), were less satisfied with their relationship (OR = 0.93, 95% CI: 0.88–0.98), were less likely to have a partner with 12 or more years of education (OR = 0.57, 95% CI: 0.34–0.96), and were more likely to have a partner scoring high on the EPDS (OR = 1.08, 95% CI: 1.02–1.13) compared to (expectant) fathers in the *not depressed* class.

**Table 3:** Predictors of latent depression class membership.

	**Anhedonic vs. not depressed**			**Anxious-worried vs. not depressed**			**Depressive vs. not depressed**		
	**OR**	**95% CI**	***p*-value**	**OR**	**95% CI**	***p*-value**	**OR**	**95% CI**	***p*-value**
Age	1.01	0.95–1.07	0.854	**0.91**	**0.85–0.98**	**0.017**	1.02	0.91–1.14	0.789
**Years of school education**									
<12 years (reference)	1.00			1.00			1.00		
12 or more years	0.72	0.44–1.16	0.171	**0.51**	**0.31–0.84**	**0.009**	0.61	0.23–1.61	0.315
**Children below age 14 in household**									
No (reference)	1.00			1.00			1.00		
Yes	1.15	0.65–2.03	0.627	0.75	0.38–1.51	0.425	**2.67**	**1.08–6.59**	**0.033**
Body Mass Index	0.97	0.91–1.03	0.283	**1.07**	**1.01–1.13**	**0.016**	1.02	0.90–1.16	0.748
**Physical activity**									
Less than once a week (reference)	1.00			1.00			1.00		
1–2 times a week	0.98	0.60–1.60	0.950	1.00	0.53–1.90	0.998	1.98	0.67–5.90	0.219
3 or more times a week	**0.48**	**0.27–0.85**	**0.012**	1.65	0.89–3.05	0.110	0.95	0.26–3.44	0.941
Perceived social support	**0.94**	**0.92–0.97**	** <0.000**	**0.94**	**0.91–0.97**	** <0.000**	**0.93**	**0.89–0.99**	**0.011**
Relationship satisfaction	**0.93**	**0.88–0.98**	**0.006**	0.96	0.90–1.02	0.213	**0.87**	**0.77–0.98**	** <0.027**
Inequitable distribution of domestic work	1.12	0.92–1.36	0.263	1.02	0.80–1.28	0.903	1.24	0.84–1.85	0.279
Expectant mother's age	1.01	0.94–1.09	0.715	**1.11**	**1.02–1.21**	**0.018**	0.98	0.87–1.10	0.711
**Expectant mother's school education**									
<12 years (reference)	1.00			1.00			1.00		
12 or more years	**0.57**	**0.34–0.96**	**0.034**	0.89	0.52–1.53	0.682	0.81	0.30–2.21	0.680
Expectant mother's EPDS score	**1.08**	**1.02–1.13**	**0.004**	**1.07**	**1.01–1.14**	**0.016**	1.06	0.96–1.18	0.240

*OR, Odds Ratio; CI, Confidence Interval; EPDS, Edinburgh Postnatal Depression Scale. Results adjusted for smoking status and alcohol consumption. Estimates in bold font indicate statistical significance (p <0.05). N = 1,027*.

(Expectant) fathers in the *anxious-worried* class were younger (OR = 0.91, 95% CI: 0.85–0.98), were less likely to have 12 or more years of school education (OR = 0.51, 95% CI: 0.31–0.84), were more likely to have a higher BMI (OR = 1.07, 95% CI: 1.01–1.13), reported lower social support (OR = 0.94, 95% CI: 0.91–0.97), and were more likely to have a partner of older age (OR = 1.11, 95% CI: 1.02–1.21) and to have a partner scoring high on the EPDS (OR = 1.07, 95% CI: 1.01–1.14) compared to (expectant) fathers in the *not depressed* class.

(Expectant) fathers in the *depressive* class were more likely to have children below age 14 in the household (OR = 2.67, 95% CI: 1.08–6.59), reported lower social support (OR = 0.93, 95% CI: 0.89–0.99), and were less satisfied with their relationship (OR = 0.87, 95% CI: 0.77–0.98) compared to (expectant) fathers in the *not depressed* class.

### Stability of Latent Depression Profile Class Membership

Table 4 shows the probabilities of transitioning from a particular latent depression profile class during pregnancy to another class at 8 weeks postpartum (T2). Stability in the depression profile was highest in the *not depressed* class. (Expectant) fathers allocated to this class during pregnancy had a probability of 0.884 of still being classified as *not depressed* at 8 weeks postpartum. For the other classes, probabilities that an (expectant) father would transition to the *not depressed* class ranged between 0.265 (*depressive*) and 0.549 (*anhedonic*). Stability in the depression profile was lowest in the *depressive* class. (Expectant) fathers allocated to this class during pregnancy had a probability of 0.337 of remaining *depressive* at 8 weeks postpartum (T2). For the other classes, probabilities of transitioning to the *depressive* class ranged between 0.004 (*not depressed*) and 0.027 (*anxious-worried*).

**Table 4:** Latent transition probabilities.

		**Latent depression profile class at 8 weeks postpartum**			
		**Not depressed (*n* = 789, 77%)**	**Anhedonic (*n* = 138, 13%)**	**Anxious-worried (*n* = 79, 8%)**	**Depressive (*n* = 21, 2%)**
	Not depressed (*n* = 677, 66%)	**0.884**	0.083	0.028	0.004
Latent depression profile class during pregnancy	Anhedonic (*n* = 178, 17%)	0.549	**0.420**	0.010	0.021
	Anxious-worried (*n* = 142, 14%)	0.492	0.061	**0.420**	0.027
	Depressive (*n* = 30, 3%)	0.265	0.118	0.281	**0.337**

*Every transition probability describes the probability of class membership 8 weeks postpartum (columns) given class membership during pregnancy (rows). Transition probabilities in bold font correspond to membership in the same latent status at both times. Darker shadings indicate higher probabilities. N = 1,027*.

## Discussion

This prospective population-based cohort study estimated prevalence rates of paternal perinatal depression symptoms, identified paternal perinatal depression profiles as well as predictors of those latent depression profiles, and estimated latent transition probabilities in the identified subgroups during the perinatal period. Our data revealed four main findings. First, about one in 10 expectant fathers reported depression symptoms; lower rates were reported at 8 weeks postpartum. Second, four qualitatively different depression profiles exist within the study population, with two-thirds of the (expectant) fathers experiencing no or few depression symptoms. Third, perceived social support and relationship satisfaction may be protective against paternal depression symptoms. Fourth, expectant fathers with absence of depression are very unlikely to develop symptoms at postpartum; those with symptomatology during pregnancy are likely to experience improvement or even remission from depression symptoms.

We found that rates of paternal depression symptoms decreased from T1 to T2, with 9% during pregnancy (i.e., 5% likely minor and 4% likely major depression) and 5% at 8 weeks postpartum (i.e., 2% likely minor and 3% likely major depression). These prevalence rates are slightly lower than what was found as an overall rate in a previous meta-analysis (19). However, the meta-analysis found that national origin of the study accounted for considerable variability in depression rates of fathers. While (expectant) fathers in the United States reported somewhat higher depression rates (14.1% on average), the average rate of the international studies was 8.2%, and thus rather similar to our results. Further, a more recent meta-analysis found an overall prevalence rate of paternal perinatal depression of 8% (51). Likewise, in line with these meta-analyses' results suggesting that the first 3 months postpartum are characterized by especially low depression rates (19, 51), we found lower prevalence rates at 8 weeks postpartum compared to the prior assessment during pregnancy. Still, a recent study among first-time fathers identified a subgroup of fathers whose depressive symptoms were highest at 1 year postpartum (52). Therefore, data from a longer follow-up period are needed before more definite conclusions can be drawn.

Further, four latent depression profiles emerged: most (expectant) fathers did not exhibit any depression symptoms (*not depressed*), whereas some reported mainly the absence of joy (*anhedonic*) and some experienced mainly self-blame and worries (*anxious-worried*). The *depressive* profile was characterized by endorsement to most symptoms of perinatal depression. Tuohy and McVey conducted a principal component analysis of the EPDS in recent mothers and found that the scale encompasses three dimensions: depression, anhedonia, and anxiety (53). Our own findings add and complement these prior findings gained from a traditional variable-centered approach with a person-centered approach proposing that the EPDS appears to reflect important dimensionality both toward symptomatology as well as toward inherent latent subgroups.

Even though we found some differential predictors of the respective depression profiles, several of the potentially modifiable predictors displayed similar patterns. All three depression profiles with at least some depression symptomatology (i.e., the *anhedonic, anxious-worried*, and *depressive* class) reported lower social support and individuals in the *anhedonic* and the *depressive* class were less satisfied with their relationships. Hence, perceived social support and relationship satisfaction served as protective factors; this finding is similar to a previous study on emotional distress in couples during pregnancy (54). Maternal depression, on the other hand, served as a risk factor for both the *anhedonic* and *anxious-worried* class, corresponding to results of a meta-analysis estimating moderate maternal-paternal depression symptom correlation (19). Interestingly, (expectant) fathers with an *anhedonic* profile were less likely to engage in physical activity three or more times a week compared to those with absence of depression symptoms, whereas this association was not found in those with an *anxious-worried* or *depressive* profile. Moreover, having children below the age of 14 in the same household prior to the index baby increased the risk for being categorized into the *depressive* profile, but not into the *anhedonic* or *anxious-worried* profile. Although our data do not allow causal inferences to be made, this may have implications for the development of future mental health interventions tailored to the respective paternal depression profile.

Our results showed differential transitioning or stability patterns in profile membership during the perinatal period. The *depressive* profile was the least stable (as underlined by the fact that fewer fathers scored above the EPDS major and minor cut-offs during the postpartum period): the probability to be classified as *depressive* at 8 weeks postpartum, if one was already classified as *depressive* during pregnancy was 34%. In contrast, the probability of remaining *not depressed* was 88%. Transitioning into the *depressive* profile at 8 weeks postpartum was very unlikely. This is entirely in line with findings from studies on *maternal* perinatal depression showing that one of the most important risks factors for postpartum depression is depressive symptomatology during pregnancy (6, 7). Interestingly, the *anhedonic* and *anxious-worried* profile exhibited somewhat parallel courses and there were practically no transitions between these two profiles. Rather, members of these two profiles either stayed in the same profile or changed to the *not depressed* profile, suggesting that they indeed represent qualitatively different depression profile patterns.

These findings suggest that the ideal time to initiate prevention measures is during the prenatal period, even in individuals who have sub-clinical symptom levels. Furthermore, it may be speculated that the *anhedonic* and *anxious-worried* profiles are mildly symptomatic precursors of depression, and thus may represent target groups for prevention with low threshold symptomatology. However, we are unable to validate the clinical significance of depression symptoms in these groups.

## Strengths and Limitations

To our knowledge, this prospective population-based cohort study is the first study to identify paternal perinatal depression profiles together with their predictors and changes during the perinatal period. We used state-of-the-art categorical latent variable modeling; this technique allowed us to use all available data of a large sample of (expectant) fathers, to classify them into meaningful (a priori unknown) subgroups representing unobserved heterogeneity in the larger population, and to identify underlying covariates or mechanisms which cause the heterogeneity. Despite our large sample size, some potentially important predictors (e.g., socio-economic status) could not be taken into account because of very small cell sizes in the joint distribution. Further, transition probabilities could not be predicted due to the same reason. As a result, parameter estimates would not have been trustworthy. Another limitation is the lack of measurements after 8 weeks postpartum, as previous literature suggests that paternal depression could have a late onset (19). Also, the assessment of depression symptoms was based on self-report and depression symptoms may be under-reported due to social desirability. Even though the EPDS has been validated in numerous studies, this has been done primarily in women; further, it is well-established that EPDS is a screening instrument rather than a clinical diagnostic tool. Finally, selection bias may have limited the generalizability for the whole population of (expectant) fathers. The high proportion of higher-educated participants may also be explained by characteristics of the recruitment setting. The Dresden population includes a large number of university students and employees. Further, persons with higher rather than lower socioeconomic status may be more likely to take part in hospital information sessions and birth preparation courses.

## Conclusion and Directions for Future Research

This prospective population-based cohort study is the first study to identify paternal perinatal depression profiles together with their predictors and changes during the perinatal period. To this end we used state-of-the-art categorical latent variable modeling to classify our large sample of (expectant) fathers into meaningful subgroups. The findings from this study suggest that future research is warranted to examine whether the identified paternal depression profiles have differential outcomes, particularly in the context of person-centered prevention and intervention strategies. In a similar vein, it would be of great relevance to determine whether the *depressive* profile in fact represents a group in need of clinical treatment. Finally, future studies ought to investigate longitudinal trajectories of paternal depression, taking into account additional measurement points as well as modifiable risk factors. Following up the study's participants, we will be eventually able to do so with the DREAM study applying growth mixture modeling (GMM).

## Data Availability Statement

The datasets presented in this article are not readily available because of legal and ethical constraints. Public sharing of participant data was not included in the informed consent of the study. Requests to access the datasets should be directed to Susan Garthus-Niegel, susan.garthus-niegel@uniklinikum-dresden.de.

## Ethics Statement

The DREAM study has been reviewed and approved by the Ethics Committee of the Faculty of Medicine of the Technische Universität Dresden (No: EK 278062015), and all Participants provided their written informed consent to participate in this study.

## Author Contributions

This study was conceived and research questions developed by SG-N and SB. AS and SB performed the statistical analysis. Data were interpreted by SG-N, AS, PK, SH, FD, and SB. SG-N, AS, and SB wrote the manuscript. SG-N has acquired the funding and been responsible for conception and design of the DREAM study as well as the coordination and supervision of the (ongoing) data collection. All authors contributed to the article and approved the submitted version.

## Conflict of Interest

The authors declare that the research was conducted in the absence of any commercial or financial relationships that could be construed as a potential conflict of interest.
