# Supplementary material for: Predictors and Changes in Paternal Perinatal Depression Profiles—Insights From the DREAM Study
Source: Front Psychiatry. 2020 Oct 29;11:563761. doi: 10.3389/fpsyt.2020.563761 (PMC7658469; doi:10.3389/fpsyt.2020.563761)
Supplement: Supplementary file 1 [file Data_Sheet_1.PDF]

```
Title:
  Latent transition analysis for paternal perinatal depression profiles

Data:
  File is dream_lca.dat;

Variable:
  Names are
     id epds_1 epds_2 epds_3 epds_4 epds_5 epds_6 epds_7 epds_8 epds_9
     epds_10 epds_1_2 epds_2_2 epds_3_2 epds_4_2 epds_5_2 epds_6_2 epds_7_2
     epds_8_2 epds_9_2 epds_10_2 alter_m bild_m kinder_im_hh bmi rauchen
     alkohol_cat sport partner_qualitaet soz_unter T1_odlq_30 odlq_neu
     alter_w bild_w epds_w flow_t1_analysis flow_t2 vaeter_lca;

  Usevar are
     alter_w alter_m partner_qualitaet soz_unter epds_w odlq_neu bmi
     bild_m kinder_im_hh bild_w rau_frueher rau_akt alk_1_2 alk_3_7
     alk_8 sport_1_2 sport_3
     epds_1d epds_2d epds_3d epds_4d epds_5d epds_6d
     epds_7d epds_8d epds_9d epds_10d epds_1d2 epds_2d2
     epds_3d2 epds_4d2 epds_5d2 epds_6d2 epds_7d2
     epds_8d2 epds_9d2 epds_10d2;

  Categorical are epds_1d-epds_10d epds_1d2-epds_10d2;
  ! epds_1d-epds_10d are dichotomized EPDS items assessed during pregnancy
  ! epds_1d2-epds_10d2 are dichotomized EPDS items assessed at 8 weeks postpartum

  Classes are c1(4) c2(4);
  ! Separate latent class models indicated four latent classes

  Useobservations are vaeter_lca == 1;
  Missing are all (-9999);

Define:
  ! Smoking, alcohol use and physical activity are dummy-coded
  rau_frueher = 0;
  IF (rauchen == 1) then rau_frueher = 1;
  rau_akt = 0;
  IF (rauchen == 2) then rau_akt = 1;
  alk_1_2 = 0;
  IF (alkohol_cat == 1) then alk_1_2 = 1;
  alk_3_7 = 0;
  IF (alkohol_cat == 2) then alk_3_7 = 1;
  alk_8 = 0;
  IF (alkohol_cat == 3) then alk_8 = 1;
  sport_1_2 = 0;
  IF (sport == 1) then sport_1_2 = 1;
  sport_3 = 0;
  IF (sport == 2) then sport_3 = 1;

  ! Dichotomization of EPDS items assessed during pregnancy
  epds_1d = 0;
  IF (epds_1 > 0) then epds_1d = 1;
  epds_2d = 0;
  IF (epds_2 > 0) then epds_2d = 1;
  epds_3d = 0;
  IF (epds_3 > 1) then epds_3d = 1;
  epds_4d = 0;
  IF (epds_4 > 1) then epds_4d = 1;
  epds_5d = 0;
  IF (epds_5 > 1) then epds_5d = 1;
  epds_6d = 0;
  IF (epds_6 > 1) then epds_6d = 1;
  epds_7d = 0;
  IF (epds_7 > 1) then epds_7d = 1;
  epds_8d = 0;
  IF (epds_8 > 1) then epds_8d = 1;
  epds_9d = 0;
```

```
  IF (epds_9 > 0) then epds_9d = 1;
  epds_10d = 0;
  IF (epds_10 > 0) then epds_10d = 1;

  ! Dichotomization of EPDS items assessed at 8 weeks postpartum
  epds_1d2 = 0;
  IF (epds_1_2 > 0) then epds_1d2 = 1;
  epds_2d2 = 0;
  IF (epds_2_2 > 0) then epds_2d2 = 1;
  epds_3d2 = 0;
  IF (epds_3_2 > 1) then epds_3d2 = 1;
  epds_4d2 = 0;
  IF (epds_4_2 > 1) then epds_4d2 = 1;
  epds_5d2 = 0;
  IF (epds_5_2 > 1) then epds_5d2 = 1;
  epds_6d2 = 0;
  IF (epds_6_2 > 1) then epds_6d2 = 1;
  epds_7d2 = 0;
  IF (epds_7_2 > 1) then epds_7d2 = 1;
  epds_8d2 = 0;
  IF (epds_8_2 > 1) then epds_8d2 = 1;
  epds_9d2 = 0;
  IF (epds_9_2 > 0) then epds_9d2 = 1;
  epds_10d2 = 0;
  IF (epds_10_2 > 0) then epds_10d2 = 1;

CENTER soz_unter epds_w odlq_neu partner_qualitaet bmi alter_m alter_w
 (GRANDMEAN);

Analysis:
  Type = mixture ;
  Starts = 1000 10;

Model:
  %OVERALL%
  c2 on c1;
  c1 on bild_m kinder_im_hh bild_w alter_m bmi
  rau_frueher rau_akt alk_1_2 alk_3_7 alk_8 sport_1_2 sport_3
  soz_unter partner_qualitaet odlq_neu epds_w alter_w;
  ! Covariates predict latent class membership during pregnancy
  ! Comparisons of different LTA models support the assumption of ...
  ! ... measurement invariance of latent classes over time

Model c1:
  %C1#1%
  [epds_1d$1] (1);
  [epds_2d$1] (2);
  [epds_3d$1] (3);
  [epds_4d$1] (4);
  [epds_5d$1] (5);
  [epds_6d$1] (6);
  [epds_7d$1] (7);
  [epds_8d$1] (8);
  [epds_9d$1] (9);
  [epds_10d$1] (10);
  %C1#2%
  [epds_1d$1] (11);
  [epds_2d$1] (12);
  [epds_3d$1] (13);
  [epds_4d$1] (14);
  [epds_5d$1] (15);
  [epds_6d$1] (16);
  [epds_7d$1] (17);
  [epds_8d$1] (18);
  [epds_9d$1] (19);
  [epds_10d$1] (20);
  %C1#3%
  [epds_1d$1] (21);
```

```
  [epds_2d$1] (22);
  [epds_3d$1] (23);
  [epds_4d$1] (24);
  [epds_5d$1] (25);
  [epds_6d$1] (26);
  [epds_7d$1] (27);
  [epds_8d$1] (28);
  [epds_9d$1] (29);
  [epds_10d$1] (30);
  %C1#4%
  [epds_1d$1] (31);
  [epds_2d$1] (32);
  [epds_3d$1] (33);
  [epds_4d$1] (34);
  [epds_5d$1] (35);
  [epds_6d$1] (36);
  [epds_7d$1] (37);
  [epds_8d$1] (38);
  [epds_9d$1] (39);
  [epds_10d$1] (40);

Model c2:
  %C2#1%
  [epds_1d2$1] (1);
  [epds_2d2$1] (2);
  [epds_3d2$1] (3);
  [epds_4d2$1] (4);
  [epds_5d2$1] (5);
  [epds_6d2$1] (6);
  [epds_7d2$1] (7);
  [epds_8d2$1] (8);
  [epds_9d2$1] (9);
  [epds_10d2$1] (10);
  %C2#2%
  [epds_1d2$1] (11);
  [epds_2d2$1] (12);
  [epds_3d2$1] (13);
  [epds_4d2$1] (14);
  [epds_5d2$1] (15);
  [epds_6d2$1] (16);
  [epds_7d2$1] (17);
  [epds_8d2$1] (18);
  [epds_9d2$1] (19);
  [epds_10d2$1] (20);
  %C2#3%
  [epds_1d2$1] (21);
  [epds_2d2$1] (22);
  [epds_3d2$1] (23);
  [epds_4d2$1] (24);
  [epds_5d2$1] (25);
  [epds_6d2$1] (26);
  [epds_7d2$1] (27);
  [epds_8d2$1] (28);
  [epds_9d2$1] (29);
  [epds_10d2$1] (30);
  %C2#4%
  [epds_1d2$1] (31);
  [epds_2d2$1] (32);
  [epds_3d2$1] (33);
  [epds_4d2$1] (34);
  [epds_5d2$1] (35);
  [epds_6d2$1] (36);
  [epds_7d2$1] (37);
  [epds_8d2$1] (38);
  [epds_9d2$1] (39);
  [epds_10d2$1] (40);

Output:
```

```
  Tech1 svalues;
```
